# Effect of cough assist device on blood gasses and period of mechanical ventilator for pneumonic children with general hypotonia

**DOI:** 10.1186/s43161-021-00049-5

**Published:** 2021-10-26

**Authors:** Raheel Sanad, Shimaa Mohamed Refaat, Bassant Meligy, Faten Hassan Abdelazeim

**Affiliations:** 1 Cairo University Specialized Pediatric Hospital (CUSPH), Cairo, Egypt; 2 Faculty of Physical Therapy, Cairo University, Cairo, Egypt

**Keywords:** Cough assist device, Mechanical ventilator, Children with pneumonia, Blood gasses, General hypotonia

## Abstract

**Background:**

Cough assist devices play an important role with patients in the intensive care unit (ICU), particularly those with neuromuscular diseases which had abnormal muscle tone. It was previously thought to be the main technique for improving cough efficacy, which aids in weaning from mechanical ventilation and improves patient outcomes. So, we selected randomly the odd numbers from Abo El-Reesh hospital records, and 30 children of both sexes with pneumonia were selected, aged from 6 months to 3 years, who were selected from intensive care unit: fifteen children were the control group, who received the selected physical therapy program (postural drainage, percussion and resist diaphragm), and another fifteen children were the study group, who received previous physical therapy program, which was selected, and cough assist device.

**Results:**

This study revealed the effect of cough assist device on blood gasses and the period of mechanical ventilator on children with pneumonia and general hypotonia. There was a significant decrease in pH, PaCO_2_, and HCO_3_ post treatment compared with that pretreatment in the study and control groups (*p* < 0.05). The percentage of decrease in pH, PaCO_2_, and HCO_3_ in study group A were 0.4, 38.26, and 30.28% respectively while that in the control group were 0.4, 32.56, and 25.48% for pH, PaCO_2_, and HCO_3_ respectively. There was a significant increase in PaO_2_ post treatment compared with that pretreatment in the study and control groups (*p* < 0.001). The percentage of increase in PaO_2_ in the study group was 32.13% and that in control group was 30.09%. There was no significant difference in pH, PaCO_2_, and HCO_3_ between both groups pre-treatment (*p* ˃ 0.05). Also, there was no significant difference between groups post treatment (*p* ˃ 0.05). The median (IQR) period of mechanical ventilation in the study group was 5 (7, 4) days while that in control group was 8 (18, 5) days. There was a significant decrease in period of mechanical ventilator of the study group compared with that of control group (*p* = 0.03).

**Conclusion:**

The cough assist device and physical therapy program were selected for children in the intensive care unit which have the same effect on arterial blood gasses as they optimize pH, PO_2_, PCO_2_, and HCO_3_, but the cough assist device helps in accelerating weaning of mechanical ventilator so as decrease the period of mechanical ventilator.

## Background

Pneumonia is an acute inflammation of the lower respiratory tract that was once thought to be a main cause of death worldwide. It was a significant factor that contributed to the death of children, along with other factors such as malnutrition. The most common infecting organisms in children are respiratory viruses and *Streptococcus* pneumonia [1]. There is also community-acquired pneumonia (CAP), which is common among children all over the world, but its incidence and mortality rate are significantly higher in poor countries than in the developed world. It is estimated that approximately 151 million new episodes occur each year among Third World children aged 5 years, resulting in an increase in incidence of 0.29 episodes per child-year, and a mortality rate of 1.3 or 2.6% or 1, 2, or more than 2 million per year is reported [2].

Ventilator-associated pneumonia (VAP) is a respiratory disorder that occurs 48–72 h after endotracheal canalization, and it is distinguished by the presence of a replacement or progressive infiltrate, general infection symptoms such as fever, changing in white somatic cell count, and disturbance at liquid body substance characteristics [3]. An active breathing technique is performed by the patient with the assistance of physical therapist to help remove the sticky secretions in the lungs. The active breathing technique (ACBT) refers to techniques which include breathing exercises to enhance the effectiveness of a cough, removing sticky secretions and enhancing ventilation process. ACBT has three phases: breathing control, deep breathing exercises or chest expansion exercises, and forced expiratory technique (F.E.T). A manual technique (MT) or positive pressure can be added when indicated, to create a complicated technique to increase the removal of sticky secretions from the lungs [4].

Hypotonia, which refers to reducing the tone in the muscles in all the body, can be discovered either at birth or later in life (neonate or childhood). Because of its presence as a feature of many disorders, it is difficult to detect its occurrence; there are many exceptions to this rule, such as in cases of congenital myotonic dystrophy or severe cases of congenital myopathies. Patients may present with general hypotonia associated with difficulties in swallowing and respiratory distress; there are many types of hypotonic diseases such as spinal muscle atrophy with general hypotonia and hemidiaphragmatic paresis; so, it causes severe complicated respiratory problems [5]. Cough can be affected in patients with affection of the neuromuscular system, such as neuromyopathy and hypotonic children; so, the techniques that enhance the cough efficacy are very important, because they speed up the weaning from mechanical ventilation and improve patient outcomes. Cough augmentation techniques involve glottal closure before coughing, and mechanical ventilator invasive tubes do not permit glottal closure, so it had a bad effect on cough efficacy [6].

Mechanically assisted coughing techniques, such as cough assist device, create a positive pressure and increase the pulmonary insufflation, followed immediately by an exsufflation by using negative pressure. This abrupt changing from positive to negative pressure creates air flows during the exsufflation phase, which are able to remove the sticky sputum in the wall of the alveoli [7]. Respiratory physiotherapy is more than just clearing the sputum as possible; it also can move the peripheral secretions which are found at the alveoli to more central regions where they can be expelled through coughing or suctioning in this way; because the patients on mechanical ventilation are exposed to secretion retention and low cough peak flow, cough assist device is very beneficial to those patients as it helps in the expulsion of these secretions [8].

## Methods

### Sample

We randomly selected the odd numbers of Abo El-Reesh hospital records of the patients and chose 30 intubated children with pneumonia, of both sexes, which will participate in this study.

Their age ranged between 6 months and 3 years old; they have been divided into two groups of equal number:

- Group A (study group): 15 children who received cough assist device and received traditional techniques of respiratory physical therapy.
- Group B (control group): 15 children who received only traditional techniques of respiratory physical therapy.

### Sample size

Sample size calculation was performed prior to the study using G*POWER statistical software (version 3.1.9.2; Universital Kiel, Germany) and revealed that the required size of each group was 15.

The calculations were made using *α* = 0.05, *β* = 0.2, and effect size = 1.1.

### Inclusion criteria

Children with the following criteria will be enrolled in this study:

- Age: 6 months to 3 years old
- Children were diagnosed with pneumonia
- Children were intubated on a mechanical ventilator (invasive ventilation with a tube inserted into the patient’s airway with positive pressure)
- Hemodynamically stable patient (heart rate with in normal rate)
- Patient received his prescribed medications
- Patient with hypotonia
- Patient with old pneumothorax (no chest tube, no collapsed lung)

### Exclusion criteria

Subjects had been excluded from the study, if they had any of the following:

1. Hemodynamically unstable patient
2. Patient with pneumothorax (if chest tube is present)
3. Asthmatic child
4. Patient with chest deformities
5. Patient with pleural effusion
6. Patient with diaphragmatic hernia
7. Patient with cardiac and thoracic surgery

### Instruments

A. Measurement of arterial blood gasses (ABG)

B. The cough assist device (cough assist T70; PHILIPS respironics made in USA)

### Procedure

To measure the arterial blood gasses (ABG), the blood sample taken by the nurse to record the result of (pH, PCO_2_, PO_2_, HCO_3_) every day after the session and compared the result between the first session and the final session when the child was extubated. In the control group, we used firstly modified postural drainage and applied percussion (10 min at least at the apical, anterior, middle, and basal loops of both lungs); then, we put the patient at the crock laying position and resisted the diaphragm muscle during inspiration to increase inhalation; finally, we did a passive range of motion for the patient. The duration of session was between 20 and 30 min as guided by subject fatigue and comfort, and we did one session daily; the child must be not on feeding or suction at least 2 h prior to the session.

In the study group, we added the cough assist device to the previous physical therapy program, in which it generated positive pressure which increases the pulmonary insufflation which can remove the sticky respiratory secretions; the session time was 1 h, and at the end of the session, if the child was exhausted, firstly, the patient position was in semi-recline position, and we injected saline in tube; secondly, we powered the device from the power button; the cough assist device protocol included 3–5 cycles with insufflation/exsufflation, positive starting from + 15 cm H_2_O and maximum pressure of + 40 cm, negative pressure starting from − 15 cm H_2_O and maximum − 40 cm H_26_O. The insufflation and exsufflation times were each 2–3 s, and the pause time was 1 s. The pressure for each subject was adjusted according to child age, amount of secretions, tolerance of patients, and chest auscultation through every session; then, we connected the tube of the device to the tube of the patient and pressed finish; rest periods were needed to suction secretions from the mouth, nose, or tracheostomy. The numbers of total sets were 4–6 with a duration of 30 min; we applied this device, used daily, one session at the day.

Measurement of mechanical ventilator period had been calculated (the period from the first day of intubation until the last day).

### Data analysis

Descriptive statistics and unpaired *t* test were conducted for comparison of age between both groups. Chi-squared test was carried out for comparison of sex distribution between groups. Normal distribution of data was checked using the Shapiro-Wilk test. Levene’s test for homogeneity of variances was conducted to test the homogeneity between groups. The period of mechanical ventilator was compared between groups by Mann–Whitney *U* test. Unpaired *t* test was conducted to compare the mean values of pH, PaCO_2_, PaO_2_, and HCO_3_ between groups. Paired *t* test was conducted for comparison between pre- and post-treatment in each group. The level of significance for all statistical tests was set at *p* < 0.05. All statistical analyses were conducted through the Statistical Package for Social Sciences (SPSS) version 25 for Windows (IBM SPSS, Chicago, IL, USA).

## Results

### Subject characteristics

Table 1 showed the subject characteristics of the study and control groups. There was no significant difference between both groups in the mean age and sex distribution (*p* > 0.05).

**Table 1:** Comparison of subject characteristics between the study and control groups

	Study group	Control group	***p*** value
	Mean ± SD	Mean ± SD	
**Age (months)**	26.27 ± 11.48	25.46 ± 11.88	0.85
**Sex distribution**			
**Girls**	4 (26.7%)	7 (46.7%)	0.25
**Boys**	11 (73.3%)	8 (53.3%)	

*SD* standard deviation; *p* value, probability value

### Effect of treatment on pH, PaCO_2_, PaO_2_, and HCO_3_

#### Within-group comparison

There was a significant decrease in pH, PaCO_2_, and HCO_3_ post treatment compared with that pretreatment in the study and control groups (*p* < 0.05). The percent of decrease in pH, PaCO_2_, and HCO_3_ in study group A were 0.4, 38.26, and 30.28% respectively while that in the control group were 0.4, 32.56, and 25.48% for pH, PaCO_2_, and HCO_3_ respectively. There was a significant increase in PaO_2_ post treatment compared with that pretreatment in the study and control groups (*p* < 0.001). The percent of increase in PaO_2_ in the study group was 32.13% and that in control group was 30.09% (Table 2).

**Table 2:** Median values of period of mechanical ventilator of study and control groups

	Study group	Control group		
	Median (IQR)	Median (IQR)	***U*** value	***p*** value
**Period of mechanical ventilator (days)**	5 (7, 4)	8 (18, 5)	63	0.03

*IQR* interquartile range, *U* value, Mann-Whitney test value; value; *p* value, level of significance

#### Between-group comparison

There was no significant difference in pH, PaCO_2_, and HCO_3_ between both groups pre-treatment (*p* > 0.05). Also, there was no significant difference between groups post treatment (*p* > 0.05) (Table 3).

**Table 3:** Mean pH, PaCO_2_, PaO_2_, and HCO_3_ pre- and post-treatment of the study and control groups

	Study group	Control group			
	Mean ± SD	Mean ± SD	MD	***t*** value	***p*** value
**pH**					
**Pre treatment**	7.41 ± 0.03	7.42 ± 0.02	− 0.01	− 1.13	0.26
**Post treatment**	7.38 ± 0.02	7.39 ± 0.01	− 0.01	− 1.21	0.23
**MD**	0.03	0.03			
**% of change**	0.4	0.4			
***t*** **value**	2.99	2.42			
	***p = 0.01***	***p = 0.03***			
**PaCO**_**2**_ **(mmHg)**					
**Pre-treatment**	49.66 ± 6.4	48.13 ± 7.68	1.53	0.59	0.55
**Post-treatment**	30.66 ± 5.62	32.46 ± 6.7	− 1.8	− 0.79	0.43
**MD**	19	15.67			
**% of change**	38.26	32.56			
***t*** **value**	9.98	6.28			
	***p = 0.001***	***p = 0.001***			
**PaO**_**2**_ **(mmHg)**					
**Pre-treatment**	97 ± 11.61	94 ± 10.11	− 3.4	− 0.85	0.4
**Post-treatment**	124.06 ± 9.21	104.8 ± 8.95	− 19.26	− 5.8	0.001
**MD**	− 26.66	− 10.8			
**% of change**	27.37	11.49			
***t*** **value**	− 11.6	− 6.51			
	***p = 0.001***	***p = 0.001***			
**HCO**_**3**_ **(mmol/l)**					
**Pre-treatment**	29 ± 3.12	28.85 ± 4.65	0.15	0.1	0.92
**Post-treatment**	20.22 ± 4.77	21.5 ± 3.6	− 1.28	− 0.83	0.41
**MD**	8.78	7.35			
**% of change**	30.28	25.48			
***t*** **value**	7.3	7.92			
	***p = 0.001***	***p = 0.001***			

*SD* standard deviation, *MD* mean difference; *p* value, probability

### Effect of treatment on period of mechanical ventilator

The median (IQR) period of mechanical ventilation in the study group was 5 (7, 4) days while that in control group was 8 (18, 5) days. There was a significant decrease in period of mechanical ventilation of the study group compared with that of control group (*p* = 0.03) (Table 3).

## Discussion

The aim of this study was to evaluate the effect of cough assist device and physical therapy program which was selected on the period of mechanical ventilator using arterial blood gasses scores as a method of re-evaluation. The bad effect of mechanical ventilator is reducing the patient’s work of breathing by unloading respiratory muscles in an asynchronous manner. However, it saves the patient’s life, but this increases the sputum retention and accumulation, atelectasis, ventilator-associated pneumonia, and weakness of respiratory muscle, making ventilation weaning more difficult and resulting in high morbidity and mortality rate. The accumulation of secretions affects the lung compliance and increases the work to produce normal volume of air [9]. Mechanical ventilation is a supportive therapy used to help patients who cannot maintain appropriate oxygenation or carbon dioxide elimination and patients with acute respiratory failure who need ventilator support to provide rest for the respiratory muscles and to reduce the work of breathing until the acute condition is resolved. The mobilization and clearing of the sputum from the lung during physiotherapy session play an important role in enhancing bronchial hygiene and gas exchange, which enhance the respiratory mechanics of critically ill and ventilated patients; so, this reflect on arterial blood gasses (pH, PCO_2_, PO_2_, HCO_3_) [10]. Furthermore, inadequate airway mucus clearance is linked to increased bad side effects of mechanical ventilator, such as ventilator-associated pneumonia and failure of extubation [11]. Cough assist device used in physiotherapy of critically ill mechanically ventilated patients plays very important roles which include avoiding ventilator-associated pneumonia and speeding up the weaning process of mechanical ventilator and intensive care unit (ICU) duration. Cough augmentation techniques, such as increasing the lung volume and manually and mechanically assisted cough, are used to reduce respiratory complications associated with chronic conditions, particularly neuromuscular disease [12].

Patients with neuromuscular diseases have abnormal respiratory mechanics, which lead to increase the retention of secretions on the lung and may lead to respiratory problems, such as atelectasis; in these patients, cough assist devices allow the patient to make the most of the treatment, as it enhances the physiological coughing mechanism, enhancing the removal of the accumulated secretions by positive pressure (insufflation) followed by negative pressure (exsufflation). Its use increases pulse oximetry, decreases dyspnea with appropriate tolerance and safety, and prevents atelectasis for patients [13].

On the other hand, we found that the clearing of respiratory secretions which happened during physiotherapy had an important role in increasing bronchial hygiene and gas exchange resulting in good ventilation, which improves the respiratory mechanics of patients who are critically ill and on mechanical ventilation, so this improved the arterial blood gasses (pH, PCO_2_, PO_2_, HCO_3_) [14]. So, as we saw in this study, there was a significant decrease in pH, PaCO_2_, and HCO_3_ post treatment compared with that pretreatment in the study and control groups (*p* < 0.05), and there was no significant difference in pH, PaCO_2_, and HCO_3_ between both groups pre-treatment (*p* > 0.05). Also, there was no significant difference between groups post treatment (*p* ˃ 0.05).

In this study, there was a significant decrease in the period of mechanical ventilation of the study group compared with that of the control group (*p* = 0.03). The sputum clearance techniques that we use in the control group show low result on the period of mechanical ventilator, because increasing lung secretion production (sputum) and retention of it is not the main problem for patients with NMD (neuromuscular disease) because the lung parenchyma, respiratory mucosa, and mucociliary function are not commonly affected. Also, bulbar muscle function can be affected, resulting in difficulty in swallowing, increasing lung secretion and oral secretions; so, sputum clearance is not enough for weaning process. So, impaired cough is the main problem besides the sputum retention; so, the cough assist device plays an important role in this because its mechanism (positive pressure followed by negative pressure) improves the weak cough [15, 16].

## Conclusion

Our study showed that there was no significant difference between study and control groups at the variable of arterial blood gasses (pH, PO_2_, PCO_2_, HCO_3_); on the other hand, there was a significant decrease in the period of mechanical ventilator in the study group; so, we should consider that cough assist device could be applied as an effective method in decreasing the period of mechanical ventilator.

## Data Availability

The datasets used and/or analyzed during the current study are available from the corresponding author on reasonable request.

## Abbreviations

ICU: Intensive care unit
VAP: Ventilator-associated pneumonia
ACBT: Active cycle of breathing techniques
FET: Forced expiratory technique
MT: Manual technique
